# Supplementary material for: Complexome profiling of the Chlamydomonas psb28 mutant reveals TEF5 as an early PSII assembly factor
Source: Plant Cell. 2025 Mar 17;37(6):koaf055. doi: 10.1093/plcell/koaf055 (PMC12164590; doi:10.1093/plcell/koaf055)
Supplement: koaf055_Supplementary_Data [file koaf055_supplementary_data.zip › psb28_interactive.zip!/plots/intensity/qualityPlots/peptides/COQ5A_Cre05.g245450.t1.2.html]

### Cre05.g245450.t1.2

Trivial name: COQ5A  
SeqCoverage [median]: 8.50  
Mass: 40.49 kDa  
  
 

| Sequence | MissedCleavage | Mass [Da] | wtIntensity | muIntensity | Score |
| --- | --- | --- | --- | --- | --- |
| FSAATSSGR | 0 | 882.42 | 2496.18 | 3998.80 | 131.06 |
| GAGGADAGGPVAPGR | 0 | 1208.59 | 834.28 | 819.69 | 105.98 |
| NVTDRPAALR | 1 | 1111.61 | 1638.98 | 2103.35 | 38.75 |
|  |  |  | 4969.4 | 6921.8 |  |
